# Synthesis of a grape-like conductive carbon black/Ag hybrid as the conductive filler for soft silicone rubber†

**DOI:** 10.1039/d1ra08649a

**Published:** 2022-01-05

**Authors:** Yanli Dou, Haijing Gu, Shixiang Sun, Weiguo Yao, Dongbo Guan

**Affiliations:** The Ministry of Education Key Laboratory of Automotive Material, College of Materials Science and Engineering, Jilin University Changchun 130025 PR China guandb@jlu.edu.cn

## Abstract

Conductive silicone rubber (CSR) is an outstanding stretchable conductive composite due to its excellent mechanical properties and stable conductivity. In this paper, silver nanoparticles were deposited on carbon black (CB) through a reduction reaction. The uniform dispersion of silver particles on the surface of CB as well as the grape-like branch structure of hybrid particles was formed by the condensation reaction of the hydroxyl groups of CB with (3-mercaptopropyl) trimethoxysilane (KH-590), along with the interattraction between sulfhydryl groups of KH-590 and silver ions. This sulfhydryl modified conductive carbon black/Ag hybrid filler (SMCB@Ag) avoided the high processing viscosity of CSR caused by the hydroxyl groups of CB. The percolation threshold of CSR made from SMCB@Ag was 5.5 wt% according to the percolation equation. With the addition amount of SMCB@Ag increasing to 10 wt%, the conductivity of CSR increased from 10^−5^ to about 10^1^. Moreover, the conductivity of this CSR showed excellent stability with extension of storage time and increase of stretching-recovery cycles.

## Abstract 2

Schematic of the fabrication procedure of SMCB@Ag with the grape-like structure.

## Graphical abstract

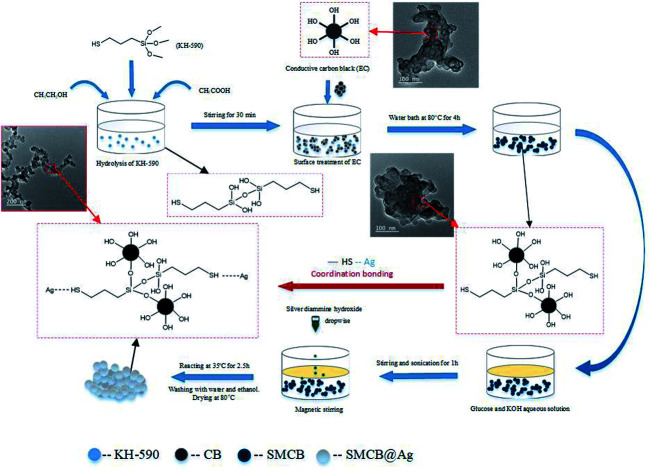

## 1 Introduction

Stretchable conductive composites (SCCs) play necessary roles in many fields due to their flexibility, recoverability and conductivity, such as electronic devices,^1,2^ electromagnetic shielding materials,^3–6^ flexible electrode materials,^7,8^ sensors^9^ and biochemical fields *etc.*^10–13^ Elastomer like silicone rubber (SR) has been widely used to build SCCs due to its super thermal stability, chemical resistance, low toxicity and low tensile modulus.^14–17^ Conductive silicone rubber (CSR) achieves electrical conductivity through the conductive network formed by the conductive fillers. The performance of CSR is strongly influenced by the type and content of conductive fillers.

Conductive fillers including metal nanoparticles,^18^ conductive carbon nanomaterials^19–21^ and conductive polymers^22–25^ can form the conductive network in matrix singly. Metal nanomaterials such as silver nanoparticles (Ag NPs) and silver nanowires (Ag NWs) have prominent conductive properties and have been fully researched and applied in microelectronics.^26^ But the high density, low flexibility and high prices of Ag NPs and Ag NWs limits the stretchability of the composites and restrains their applications in mass production and large devices.^27^ Conductive carbon nanomaterials can not only provide the conductive properties to the matrix but also strength the matrix.^28^ Among the conductive carbon nanomaterials, CB can be applied in the large-scale commercial production of conductive composites ascribed to its low price, cost-effective and durable option. However, CB has bad dispersion in polymer matrix due to its big specific surface area and low density. Lots of hydroxyl groups on the surface of CB endow it tend to be aggregated. For example, in Chen's research,^29^ CB had obvious aggregation in isoprene rubber observed in the SEM images. Zhai *et al.*^30^ first dispersed CB in 1,4-dioxane to promote the dispersion of CB in matrix, and then fabricated a conductive TPU foam. This foam has superior durability and excellent response stability.

Conductive carbon materials and metal fillers are often used together to reinforce the electrical conductivity or mechanical properties of the composites by simple mixing^31,32^ or mutual modification.^33–35^ When this hybrid filler is used to fabricate filled conductive composites, it shows good dispersibility in the matrix. Surface metallization of conductive carbon nanomaterials is a valid method to compound multiple fillers to increase the flexibility of metal particles and reduce their consumptions. Many scholars directly deposited silver particles on the surface of conductive carbon nanomaterials by the reaction between silver precursors solution and reductant solution. For example, silver trifluoroacetate solution react with the hydrazine monohydrate solution on the surface of CNT/SBS foam.^36^ Silver nitrate react with glucose or dodecylbenzene sulphonic acid (DBSA).^34,37,38^ The conductive composites filled with this prepared hybrid fillers have good conductive strain sensitivity and thermoelectric performance in practical application. When receiving an external force, the distance between filler particles in matrix will change, and the conductive network will be destroyed. With the external force removed, the particles can partly return to the original state and re-form the conductive network, making the conductive composite have high conductivity again. However, in stretchable wirings, it is necessary for conductive composites to maintain good conductivity under external force and heating conductions.^39–42^ Enhancing the interaction between carbon nanomaterials and metal particles and improving the compatibility of fillers and matrix are very effective method to improve the conductive stability of conductive composites.

The use of silane coupling agent can enhance the interaction between fillers and organic matrix. The attraction between sulfhydryl groups and metal ions is applied to absorb metals in many fields.^43^ Zhu *et al.*^44^ modified silica cryogen with (3-mercaptopropyl) trimethoxysilane (KH-590) to selectively absorb mercury in wastewater efficiently. Huang *et al.*^45^ treated the liquid metal with 3-mercaptopropionic acid through the coordination bonding of sulfhydryl groups and liquid metal and further blended it in the PU matrix modified by PDA. Zhang *et al.*^46^ immobilized the Ag NPs on the surface of poly (glycidyl methacrylate) modified with sulfhydryl (PGMA-SH). Silver nitrate was dispersed and absorbed evenly on the surface of PGMA-SH due to the attraction between sulfhydryl groups and silver ions, which causes uniform formation of silver particles on PGMA-SH. Therefore, the attraction effect of sulfhydryl groups on metals can be applied to the preparation of carbon nanomaterials and metal hybrid fillers.

In this work, KH-590 was adopted as a bridge to connect CB and Ag NPs. KH-590 was first used to treat the surface of CB by the condensation between hydroxyl groups of CB and silanol groups in KH-590. Ag NPs were deposited uniformly on the surface of KH-590 modified CB (SMCB) by Silver Mirror Reaction attributed to the absorption of sulfhydryl groups to Ag NPs. Then, the prepared filler was blended in liquid SR to fabricate the CSR. A grape-like branch structure of hybrid filler (SMCB@Ag) was observed in TEM images. The dispersion of filler in SR was shown in SEM images. The four-probe test was used to measure the static and dynamic conductivity.

## 2 Results and discussion

**Fig. 1:**
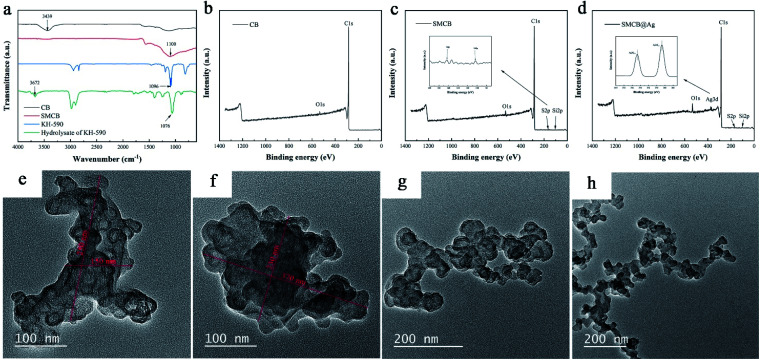
(a) FT-IR spectra of KH-590, hydrolysate of KH-590, CB and SMCB. XPS spectra of (b) CB, (c) SMCB and (d) SMCB@Ag. TEM images of (e) CB, (f) SMCB, (g) CB@Ag and (h) SMCB@Ag.

Fig. 1a shows the FT-IR spectra of KH-590, hydrolysate of KH-590, pure CB and SMCB. By comparing KH-950 and hydrolysate of KH-590, 3672 cm^−1^ corresponding to hydroxyl groups appears in hydrolysate of KH-590, that is because silane of KH-590 is hydrolyzed to silanol. The characteristic peak of Si–O–C at 1096 cm^−1^ of KH-590 shifted to Si–O–Si at 1076 cm^−1^ in hydrolysate of KH-590, due to the formation of the oligomer through self-condensation between the hydroxyl groups of hydrolytic KH-590.^47^ For the pure CB, a characteristic peak is observed at 3439 cm^−1^ due to the stretching vibration of hydroxyl groups on the surface of CB. The disappearance of hydroxyl group in SMCB demonstrates that the dehydration condensation reaction between hydroxyl groups of CB and hydrolysate of KH-590. The characteristic peak at 1100 cm^−1^ is the combined effect of Si–O–Si, Si–O–C and C–O stretching vibration in SMCB spectrum. The disappearance of hydroxyl groups of CB and the enhancement of characteristic peak at 1110 cm^−1^ in SMCB both proved the successful introduction of KH-590 on the surface of CB.

In order to further clarify the change in chemical structure, Fig. 1b–d show the XPS spectrum of CB, SMCB and SMCB@Ag. Compared with CB, the XPS spectra of SMCB and SMCB@Ag showed new peaks Si_2p_, S_2p_ and Ag_3d_. The binding energy peaks at 163.3 eV and 103.7 eV correspond to the S_2p_ and Si_2p_ in SMCB represent the introduce of KH-590 on the surface of CB in Fig. 1c.^48,49^ From the high-resolution XPS spectrum of Ag_3d_ (Fig. 1d), it can be seen that the peaks with binding energy of 368.7 eV and 374.7 eV arise from the Ag_3d_ core, indicating the characteristic peaks of zero-valent Ag, demonstrating that silver exists in the state of metal.^50^

The morphology of CB, SMCB, CB@Ag and SMCB@Ag are shown in Fig. 1e–h. The CB particles show flakes or clusters instead of grape-like shape due to the aggregation. The average diameter of pure CB flakes is about 200 nm, while it reaches about 350 nm after treated by KH-590, that is because CB particles were attracted on oligomers produced by the hydrogen bonds between hydroxyl groups of CB particles and oligomers, and then the covalent linkage is formed between them through the dehydration reaction, which makes more CB particles aggregate together. CB@Ag and SMCB@Ag appears the grape-like branch structure after deposition of silver, the aggregate CB particles are separated by the repelling effect of silver deposited on the surface of CB. SMCB@Ag has a finer branched structure compared with CB@Ag. The main reason for the difference in the microstructure of CB@Ag and SMCB@Ag is that the introduction of sulfhydryl groups on the surface of CB attracts more silver ions on the surface of CB and improves the uniform dispersion of silver ions. Because of the attraction of sulfhydryl groups to silver, SMCB will be deposited more uniform silver, which contribute to the grape-like branch structure of SMCB@Ag.

**Fig. 2:**
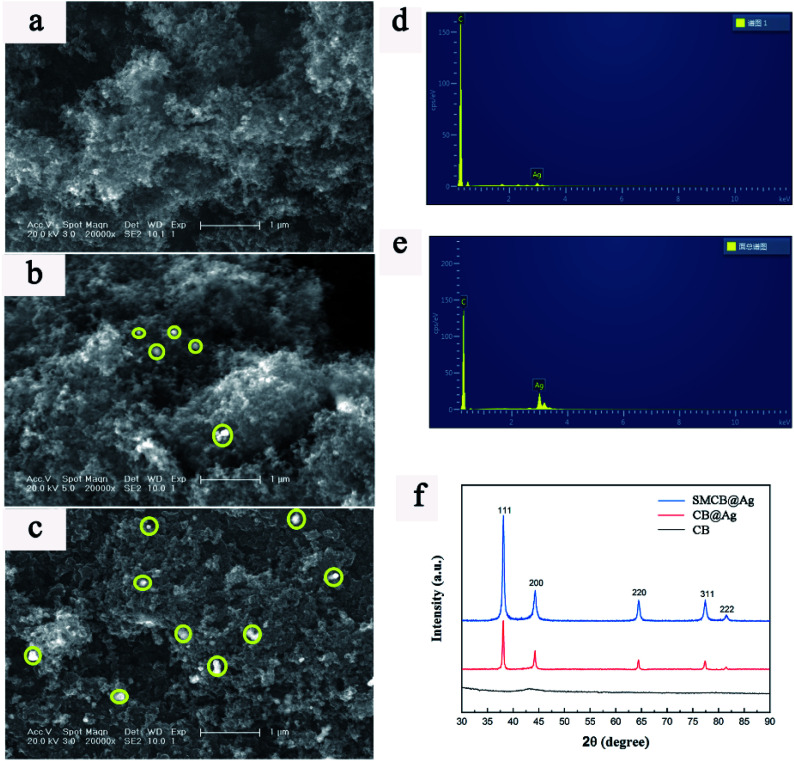
SEM images of (a) CB, (b) CB@Ag, (c) SMCB@Ag. Energy spectrums of (d) CB@Ag and (e) SMCB@Ag. (f) XRD patterns of CB, CB@Ag and SMCB@Ag.

The microstructure of the CB, CB@Ag and SMCB@Ag are shown in Fig. 2a–c. Yellow circles underline the Ag NPs deposited on the surface of CB in Fig. 2b and c. It can be clearly observed that Ag NPs has a greater uniform dispersion on the surface of CB treated with KH-590. Fig. 2d and e show the element distribution of CB@Ag and SMCB@Ag. The greater the ratio of Ag peak and the C peak, the more the Ag content. Apparently, the Ag content in SMCB@Ag is higher than that in CB@Ag. The XRD patterns of CB, CB@Ag and SMCB@Ag are displayed in Fig. 2f. The SMCB@Ag and CB@Ag both have characteristic peaks at 38.1°, 44.3°, 64.5°, 77.4° and 81.5°, which are attached to the crystal faces of (111), (200), (220), (311) and (222) of Ag. However, the SMCB@Ag has stronger characteristic peaks than CB@Ag because the introduction of sulfhydryl groups makes the reduction process of silver ions more likely to occur on the surface of SMCB instead of forming a sliver mirror on the wall of beaker. Due to the attraction of sulfhydryl groups on silver ions, the sulfhydryl groups provide reaction sites for silver reduction, causing the uniform formation of Ag NPs on the surface of SMCB.

**Fig. 3:**
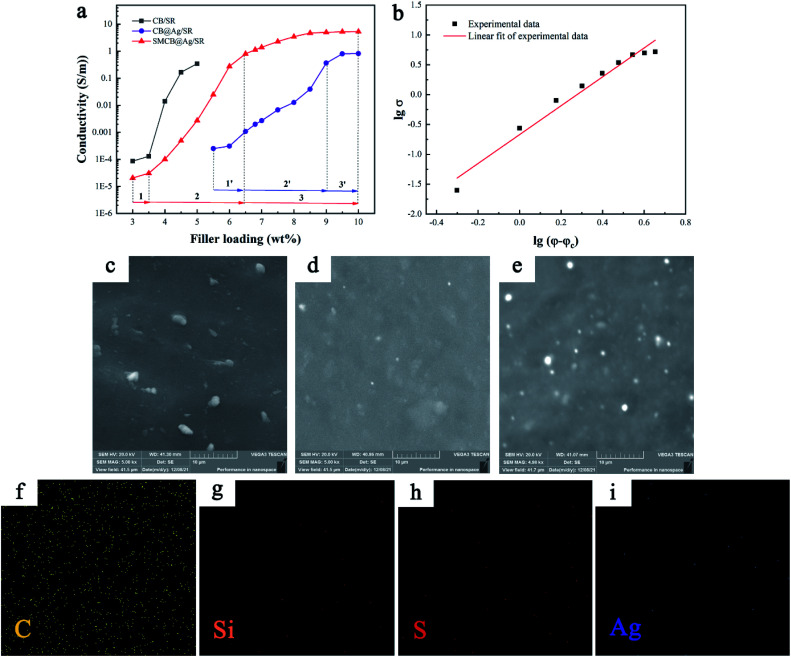
(a) The electrical conductivity of CSR filled with different fillers as a function of the filler loading. (b) The fitting line for SMCB@Ag/SR by the classical percolation theory. (c–e) SEM images of the CSR (c) 5%CB/SR, (d) 5%SMCB@Ag/SR, (e) 6%SMCB@Ag/SR. (f–i) The element mapping of C, Si, S and Ag in 6%SMCB@Ag/SR.

Fig. 3 shows the conductivity of CSR and the dispersibility of fillers in SR matrix. The viscosity of CSR made by CB increased sharply even in a very small amount of CB due to the hydroxyl groups of CB (as shown in Fig. 1a), which makes it difficult to add more CB in the SR, as a result, the maximum content of CB is only 5.0 wt% in this experiment. After the content of CB exceeds 3.5 wt%, the conductivity of CSR increases sharply. As the content increases to 5.0 wt%, the increase in conductivity is gentle. While CB@Ag and SMCB@Ag has less effect on the viscosity of CSR. In Fig. 3a, the electrical conductivity curves of CB@Ag and SMCB@Ag in terms of the filler loadings can be divided into three parts (marked in figure) respectively. In part 1 and 1′, the CSR had poor conductivity. With an increase of filler content, conductive paths gradually formed in the SR matrix. A rapid increase in electrical conductivity of CSR occurred in part 2 and 2′. In part 3 and 3′, the plateau conductivity was found in CB@Ag/SR and SMCB@Ag/SR with high filler loadings, indicating a stable conductive path was formed in the SR matrix.

By comparing the three conductivity curves, the CSR prepared with pure CB has the lowest percolation threshold about 4 wt%, demonstrating that CB has the best effect on the improvement of CSR conductivity at low content. The low amount of CB is easy to cross the barrier and form electronic transition due to the primary particles composed of graphite crystallites.^27^ However, the maximum conductivity of CSR with 5.0 wt% pure CB can only reach 0.35 S m^−1^ through experiment. As for the SMCB@Ag/SR, with the SMCB@Ag content increased to 10 wt%, the conductivity of CSR almost reached to 10 S m^−1^. The conductivity of CSR filled with SMCB@Ag increased by approximately 4 orders of magnitude at a content of 5.5 wt%, suggesting that a conductive network began to form. Since the modification of CB consumed the hydroxyl groups on the surface of CB, the maximum amount of SMCB@Ag can reach 10 wt% and the conductivity is one order of magnitude higher than the highest conductivity of CB/SR. The deposited silver particles play a key role in the improvement of conductivity with the increase of filler loadings. Besides, SMCB@Ag/SR has the better conductivity than CB@Ag/SR because in the process of silver deposition, the introduction of sulfhydryl groups had a positive influence on the reduction of silver ions. The sulfhydryl groups became deposition sites for the deposition of silver ions and increased the amount of silver deposited on the surface of CB.

Conductivity of CSR is well depended on the formation of an efficiently conductive network in the SR matrix. Normally, the percolation threshold of fillers can be fitted by the classical percolation theory.^51,52^*σ* = *σ*_0_ (*φ* − *φ*_c_)^*t*^where *φ* and *φ*_c_ are mass fraction and percolation threshold, *σ*_0_ and t are constants that are typically assigned to the plateau conductivity and scaling exponent which is used to predict the dimension of the conductive network. Usually, *t* = 2 indicates a three-dimensional conductive network appeared and *t* = 1.3 means the formation of a two-dimensional conductive network.^51^ The curve of SMCB@Ag/SR fitted according to the classical percolation theory is shown in Fig. 3b. The *φ*_c_ is 5.5 wt% and the t value is 2.4189, which is larger than 2 suggesting that a three-dimensional conductive network formed in the CSR.

Fig. 3c–e show the dispersion of CB and SMCB@Ag in CSR with 5.0 wt% CB, 5.0 wt% SMCB@Ag and 6.0 wt% SMCB@Ag. The 5%CB/Ag exhibited the aggregated CB dispersion in SR matrix. The formation of conductive network was mainly due to the formation of aggregated CB connected to each other. Compared to the 5%CB/SR, the conductive particles in 5%SMCB@Ag/SR have better dispersibility and homogeneity. The introduction of KH-590 and the formation of grape-like branch structure improve the dispersion of SMCB@Ag in SR matrix. Although the dispersion of SMCB@Ag was well, the conductive network was not fully formed with 5.0 wt% SMCB@Ag, which is below the percolation threshold. A stable and complete conductive network was formed in CSR filled with 6.0 wt% SMCB@Ag, therefore, the conductivity of CSR was abruptly improved, matching the conductive behaviour shown in Fig. 3a. The element distribution of CSR filled with 6 wt% SMCB@Ag is obtained by EDS and is shown in Fig. 3f–i. It can be clearly observed that C, Si, S and Ag dispersed uniformly in CSR. The percentage of each element is shown in Table S1.† Due to the mass fraction of filler was only 6 wt% and the Ag nanoparticles and sulfur was only a part of hybrid filler, only a very small amount of silver and sulfur can be seen in the elemental mapping. Although the very small amount of Si, S and Ag can be observed clearly, they have pretty good dispersibility, which demonstrates the good dispersibility of filler in CSR.

**Fig. 4:**
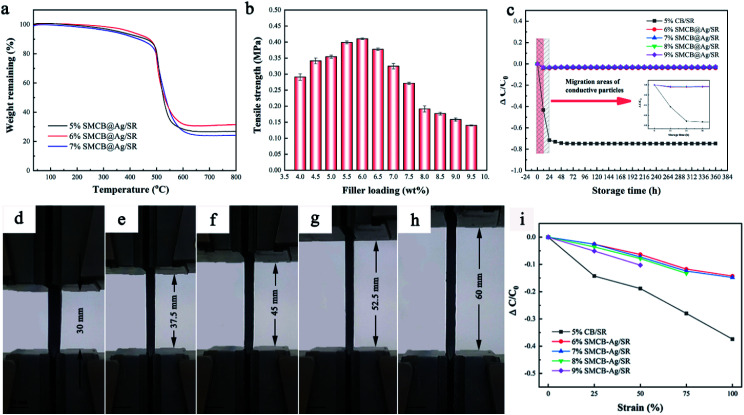
(a) TGA thermogram of CSR filled with 5.0 wt%, 6.0 wt% and 7.0 wt%SMCB@Ag. (b) The tensile strength of CSR. (c) The relationship between electrical performance and storage time. The photographs of CSR stretched up to 100% strain. (d) Pristine, (e) 25% strained, (f) 50% strained, (g) 75% strained and (h) 100% strained. (i) The relative conductivity changes of CSR (filled with 5.0 wt% CB, 6.0 wt% SMCB@Ag, 7.0 wt% SMCB@Ag, 8.0 wt% SMCB@Ag and 9.0 wt% SMCB@Ag) after different strains.

The thermal stability of CSR under different SMCB@Ag content is compared and shown in Fig. 4a. Two major weight loss can be seen for all CSR. The first weight loss could be attributed to the removal of H_2_O on the surface of CSR and degradation of a small amount of KH-590. The weight loss at 500 °C is due to the thermal degradation of SR matrix.^53^ By comparing the TGA curves of CSR with different filler loadings, it can be observed that CSR shows the best thermal stability with 6.0 wt% SMCB@Ag. The addition of fillers will damage the cross-linked network of SR, but the silane coupling agent in the filler will enhance the interaction between the fillers and the matrix. With the content increasing to 7 wt%, the SR matrix contained a lot of fillers and the cross-linking of matrix was mostly destroyed. The enhancement effect of the silane coupling agent was far from being able to compensate for the destruction of the cross-linked network. Therefore, 6%SMCB@Ag has the best thermal stability among all the CSR with SMCB@Ag added. Fig. 4b displays the tensile strength of CSR filled with different filler content. The tensile strength of CSR first increased with the increase of filler contents and then decreased. A maximum tensile strength appeared at 6.0 wt% filler content, which is similar to the results of TGA. At low content, due to the enhancement effect of nanoparticles SMCB@Ag, the tensile strength was improved.^54^ As the filler content continues to increase, the enhancement effect of the filler may no longer compensate for the decrease in mechanical properties caused by the destruction of the cross-linked network in SR.

The relative conductivity change is demonstrated by the conductivity change relative to the initial conductivity Δ*C*/*C*_0_ = (*C* – *C*_0_)/*C*_0_. The negative value of relative conductivity change indicates the decrease of conductivity with the extension of storage time. In the following experiments, the 5%CB/SR was adopted as a control group to compare with CSR prepared with SMCB@Ag due to the 5%CB/SR has the highest conductivity among CB/SR.

The relationship between electrical conductivity and storage time is shown in Fig. 4c. The conductivity of CB/SR has a sharp decrease in the first 24 h and after 24 h the conductivity gradually becomes stable. In SMCB@Ag/SR, the conductivity has no obvious change until stored for 360 h. In addition, the conductivity loss rate of SMCB@Ag/SR is controlled below 0.1, which is far lower than that of CB/SR. Fillers in conductive composites will migrate over time so that the initial formed conductive networks will be destroyed. The migration of particles (the box in Fig. 4c) decreases the conductivity of CSR. However, SMCB@Ag is hardly to migrate ascribed to the grape-like branch structure and the grafted KH-590. KH-590 in SMCB@Ag can not only connect CB with Ag particles, but also enhance the interaction between filler and matrix, which also hinders the migration of filler.

Fig. 4d–h display the photographs of CSR stretched up to 100% strain. The conductivity of CSR with different strains is shown in Fig. 4i. The conductivity of CSR reduced with the increased strains because the applied strain broke the original conductive network formed after the cure of CSR. The decline of CB/SR conductivity is more pronounced than CSR filled with SMCB@Ag because the grape-like branch structure of SMCB@Ag will extend and stretch under external force, thereby having a buffering effect resisting the external force. Meanwhile, modification of KH-590 on CB improved the interaction between the filler and SR matrix. In terms of the CSR with SMCB@Ag, with the increase of SMCB@Ag content, the conductive stability slightly decreased ascribed to the excessive fillers destroying the cross-linking of CSR. The cracks appeared in 8%SMCB@Ag/SR and 9%SMCB@Ag/SR, which results in bad conductive stability compared with 6%SMCB@Ag/SR and 7%SMCB@Ag/SR.

**Fig. 5:**
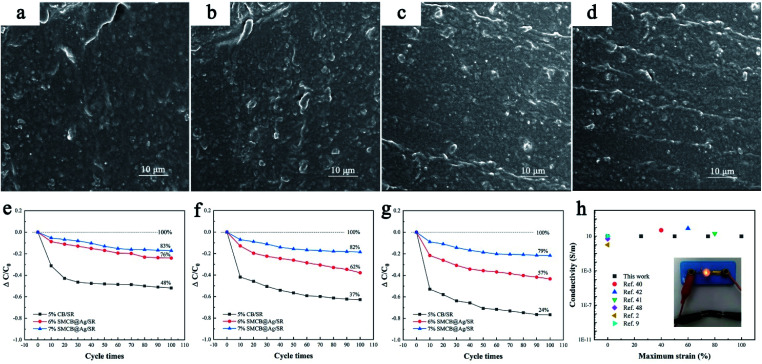
SEM images of CB/SR under 75% strain for (a) a single stretching-recovery cycle and (b) 100 cycles and 6%SMCB@Ag/SR under 75% strain for (c) a single stretching-recovery cycle and (d) 100 cycles. The conductive stability of CSR under different stretching-recovery cycles and different strains. (e) 25% strained, (f) 50% strained and (g) 75% strained. (h) Comparation of electrical properties of CSR with other conductive composites and the test image of CSR with small light bulb.

Fig. 5a–d respectively display the morphology of CB/SR and 6%SMCB@Ag/SR under 75% strain for single stretching-recovery cycle and 100 cycle times. There is an aggregation of CB in CB/SR for single stretching-recovery cycle at 75% strain and a conductive network is formed between the agglomerated CB particles. With the cycle times increasing to 100 cycles, the aggregated CB particles are separated under external force. 6%SMCB@Ag has no obvious aggregation after single stretching-recovery cycle and 100 cycles. Some conductive particles orient along the direction of applied strain, especially after 100 cycles. Regular particle orientation is observed evenly in 6%SMCB@Ag/SR. The group of conductive particles with a certain direction formed inside CSR after stretching can be called “Conductive tape”. Compared Fig. 5b with Fig. 5d, the former has no obvious “Conductivity tape” but that of 6%SMCB@Ag/SR is evenly dispersed in SR matrix, which results in a stable and uniform conductive network of SMCB@Ag/SR.

In order to further explore the cycle stability of CSR filled with SMCB@Ag and to compare it with CB/SR, the relative conductivity change of CB/SR, 6%SMCB@Ag/SR and 7%SMCB@Ag/SR at various strains from 25% to 75% for different cycle times are herein investigated, as shown in Fig. 5e–g. The same phenomenon appeared under the same strain for different cycle times that is in the first 10 stretching-recovery cycles, the conductivity of all CSR decreased sharply and after 20 cycles, the decline became gentle. Although SMCB@Ag can orient in a certain direction under a certain strain and re-form a stable conductive network, the re-form conductive network is far less than the original network so that the conductivity decreased with the stretching-recovery cycle. Based on the calculation of the ordinate value in Fig. 5e, the conductivity retention of CB/SR after 100 cycles was 48% but that of 6%SMCB@Ag/SR and 7%SMCB@Ag/SR can reach 76% and 83% respectively. Similarly, it can be concluded by Fig. 5f and g, 7%SMCB@Ag/SR showed the highest conductivity retention. Under the same strain conditions, SMCB@Ag/SR shows the better stability than CB/SR because the aggregated CB particles forming the conductive network will be separated by the external force and there is a great distance between them in CB/SR, which results in the decrease of conductivity. As for SMCB@Ag/SR, the grape-like branch structure of SMCB@Ag can extend and stretch under the external force so that the original conductive network is mostly preserved. With the increase of SMCB@Ag content, the conductive stability of CSR is improved because the increasing fillers form a more stable conductive network.

The CSR with stable conductivity is very suitable to apply in stretchable conductors,^41,42^ stretchable wirings and dielectric elastomer.^39,40^ The bulb lighting experiment of CSR is shown inside Fig. 5h. The CSR can be connected as a conductor in the circuit to light up the bulb. The comprehensive electrical properties of CSR and other conductive composite materials are summarized in Fig. 5h. The *X*-axis is the maximum strain of the conductive materials to maintain the initial conductivity, while *Y*-axis is the conductivity after corresponding strain. It can be seen that CSR in our work have a good electrical conductivity and conductive stabilities, its conductivity can reach the level of the conductive elastomer filled with carbon nanotube, carbon fiber or Cu NWs, and the cycle stability is higher than these conductive composites in the references. The specific data is listed in the ESI (Table S1†).

## 3 Experimental

### 3.1 Materials

Conductive carbon black (CB, EC600JD) was purchased from Lion King Company Japan. Ethanol and acetic acid were supplied by Tianjin Xinbote Co., Ltd., China. KH-590 was provided by Shanghai Macleans Biochemical Technology Co., Ltd., China. Silver nitrate was supplied by state-owned Yongding Chemical Plant in Beijing, China. Ammonia solution was obtained by Beijng Chemical Plant, China. Potassium hydroxide and glucose were received by National Pharmaceutical Group Chemical Reagents Co., Ltd., China. The SR matrix was bought from Shenzhen Dayou Silicone Mould Co., Ltd., China, with the base polymer (DY6840A) and the curing agent (DY6840B). Hydrogen silicone oil was obtained by Shandong Dayi Silicone Co., Ltd., China. The platinum catalyst (PC-13B, 3000 ppm) was supplied by Shanghai Neutron Star Co., Ltd., China.

### 3.2 Surface modification of CB

CB was modified with KH-590 through dehydration condensation reaction between hydroxyl groups of CB and KH-590. Typically, 0.067 g KH-590 was dispersed in 10 g ethanol and 1 ml acetic acid through vigorous stirring for 30 min. Acetic acid can promote the hydrolysis of KH-590 and maintain the stability of its hydrolysate. The pre-dispersed CB (1 g) in 30 g ethanol was then added into above suspension. The mixture was stirred for 30 min, sonicated for another 30 min, heated to 80 °C and kept for 4 h under continuous stirring. The obtained product was purified with deionized water and ethanol by the treatment of centrifugation (6500 rpm), the solution was separated and black product was obtained. After drying in an oven at 80 °C for 24 h, the target product SMCB was prepared.

### 3.3 Preparation of SMCB@Ag

A reduction process of silver nitrate occurred in this procedure. The oxidizer solution was prepared by dispersing 1 g silver nitrate in 100 g deionized water. Ammonia was added drop by drop until the solution became clear again. 1 g potassium hydroxide and 1 g glucose were dispersed in deionized water (20 g) to prepare reducing agent solution. The SMCB was added in the reducing agent solution and magnetic stirred for 30 min. The oxidizer solution was added drop by drop in the above suspension and the suspension reacted for 2.5 h at 35 °C. After the reaction, the product was purified with deionized water and ethanol to remove the remaining impurity and dried at 80 °C.

Besides, we also prepared the CB@Ag as a comparison. The experimental process was as follows: directly combine CB without being modified by KH-590 with reducing agent solution, magnetic stir for 30 min and then oxidizer solution was added dropwise. The reaction was carried out at 35 °C for 2.5 h. The purification method of the product was the same as above.

**Fig. 6:**
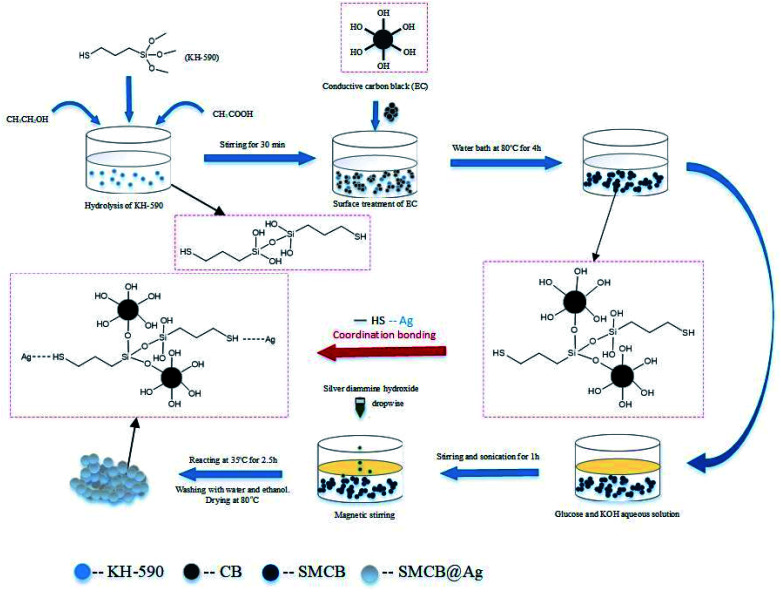
Schematic of the fabrication procedure of SMCB@Ag.

The reaction scheme of SMCB@Ag is shown in Fig. 6. The silicon methoxy groups are hydrolyzed into hydroxyl groups after KH-590 was hydrolyzed under acetic acid, which can react with the hydroxyl groups on the surface of CB. The functional groups of CB changed from hydroxyl to sulfhydryl due to the modification of KH-590. Sulfhydryl groups have a certain attraction to metal ions so that the silver ions are more likely to be absorbed and reduced on the surface of SMCB. Silver nitrate solution is added dropwise to control the reduction process and form uniform silver on the surface of SMCB. In addition, mutual exclusion between silver ions causes the formation of the grape-like branch structure of SMCB@Ag. The schematic illustration of these reactions is in Fig. S2.†

### 3.4 Preparation of SMCB@Ag/SR

The pure liquid SR was prepared by mixing base polymer (A) and curing agent (B) with a weight ratio of 1 : 1. The addition of conductive particles affects the cross-linking of SR so that it is necessary to add a certain mass of hydrogen silicone oil (H) and platinum catalyst (Pt) to ensure the complete curing of CSR. The mass ratio of each component is as follows, A : B: H : Pt is 1 : 1: 1 : 0.15. After the glue was mixed well, the conductive filler was blended into the glue, stirred for 5 min and poured into the mould or painted on the glass plate, which depends on the next performance. The amount of SMCB@Ag changed from 3 wt% to 10.0 wt% and the amount of CB was from 3.0 wt% to 5.0 wt% because the exceeded amount will result in the granular precipitated product, which is difficult to cure into specific shape according to the research of this work.

### 3.5 Characterization and measurement

Before each test, powder samples were dried in an oven at 60 °C for 24 h. The functional groups of CB, KH-590, hydrolysate of KH-590 and SMCB were characterized by Fourier infrared spectroscopy (FT-IR, TENSOR-27, Brooke Company, Germany) in the wavenumber range from 400 cm^−1^ to 4000 cm^−1^. The X-ray photoelectron spectroscopy (XPS) was measured on the ESCALAB-250Xi (Thermo Fisher) spectrometer equipped with Al Kα radiation as power source to analyse the chemical composition of CB before and after modification. Scanning electron microscopy (SEM, FEI XL30 ESEM FEG, USA) and energy dispersive X-ray spectroscopy (EDS, EDAX Genesis XM2, USA) were performed to observe the microstructure and element analysis of particles. The crystal peaks of silver on the surface of the CB were characterized by X-ray Diffractometer (XRD, 18KW/D/Max 2500pc, Rigaku Electric Co., Ltd., Japan). The particle surface morphology was observed by transmission electron microscope (TEM, JEM-2200FS, JEOL, Japan). The dispersion of filler in CSR and elemental mapping of C, Si, S and Ag was observed by Scanning Electron Microscope (VEGA3 XMU, TESCAN) and energy dispersive X-ray spectroscopy (EDS). Thermogravimetric analyzer (TGA, Beijing Hengjiu Company, China) was used to analyse the thermal decomposition properties of CSR under nitrogen atmosphere at a heating rate of 10 °C min^−1^ from 30 °C to 800 °C. The mechanical properties of CSR were measured on universal testing machine (WSM-5kN, Changchun Intelligent Instrument Equipment Co., Ltd., China) and the stretching rate was 50 mm min^−1^. The four-probe tester (ST-2258A, Suzhou Jingge Electronics Co., Ltd., China) was used to test the static resistivity and dynamic resistivity under different strain conditions of CSR. Conductivity can be calculated by the following formula:*σ* = 1/*ρ*where *σ* is the conductivity of CSR; *ρ* is the resistivity of CSR which can be directly measured.

Conductivity stability of CSR was tested by cyclic stretching-recovery treatment. The tensile strains were from 0% to 75% and the cycle times were up to 100 times. After each stretch, samples were held for 1 min, and the recovery was also kept for 1 min. The relative conductivity change was represented by Δ*C*/*C*_0_ = (*C* – *C*_0_)/*C*_0_. Where *C* is the conductivity of the CSR treated with different conditions, for example, storing at room temperature; stretching under different strains; stretching and recovering for a certain number of cycles. *C*_0_ is the initial conductivity of CSR without any treatment. The result is a negative value, demonstrating that the conductivity decreases in the static and dynamic conductivity test. The larger absolute value of Δ*C*/*C*_0_ indicates the worse conductive stability.

## 4 Conclusions

In this work, we first prepared a grape-like branch structure filler SMCB@Ag with the assistance of KH-590, and then blended the filler into SR to fabricate CSR. Due to the modification of CB with sulfhydryl groups and reduction of silver particles on the surface of CB, the viscosity of CSR prepolymer with SMCB@Ag was reduced, the highest content of SMCB@Ag can be increased to 10 wt% and the conductivity of SMCB@Ag/SR can be improved by 6 orders of magnitude, which is higher than the highest conductivity of CB/SR. The stacked grape-like branch structure can extend and stretch under the external force, which reduces the negative effect of the increasing distance of conductive fillers on the conductivity of CSR. In strain cycle stability test, the relative conductivity change of 7%SMCB@Ag/SR remained 87% after 100 cycles under 25% strain, which is much higher than that of CB/SR (48%). With the applied strain increasing to 75%, the conductivity retention of CB/SR has been reduced to 24%, but that of 7%SMCB@Ag/SR can still be maintained at 79%. Thus, the grape-like branch structure SMCB@Ag can form a more stable conductive network used in the preparation of CSR and endow CSR excellent conductive stability.

## Conflicts of interest

There are no conflicts to declare.

## Supplementary Material

RA-012-D1RA08649A-s001
