# Supplementary material for: Synthesis of a grape-like conductive carbon black/Ag hybrid as the conductive filler for soft silicone rubber
Source: RSC Adv. 2022 Jan 5;12(2):1184–93. doi: 10.1039/d1ra08649a (PMC8978839; doi:10.1039/d1ra08649a)
Supplement: RA-012-D1RA08649A-s001 [file RA-012-D1RA08649A-s001.pdf]

Electronic Supplementary Material (ESI) for RSC Advances.
This journal is © The Royal Society of Chemistry 2021

# Supplementary information

## Synthesis of grape-like conductive carbon black/Ag hybrid as the conductive filler for soft silicone rubber

Yanli Dou, [a] Haijing Gu, [a] Shixiang Sun, [a] Weiguo Yao [a] and Dongbo Guan [*a]

a The ministry of education key laboratory of automotive material, College of Materials Science and Engineering, Jilin University, Changchun PR China, 130025.

E-mail: guandb@jlu.edu.cn.

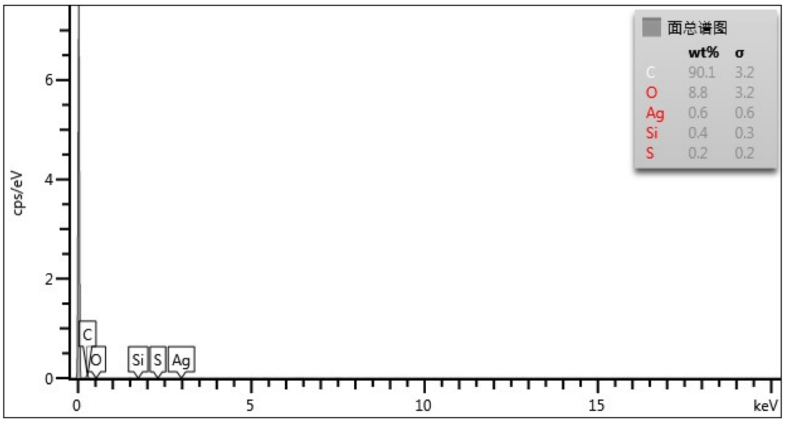


Fig. S1 The EDS spectra and elemental distribution of 6%SMCB@Ag/SR..

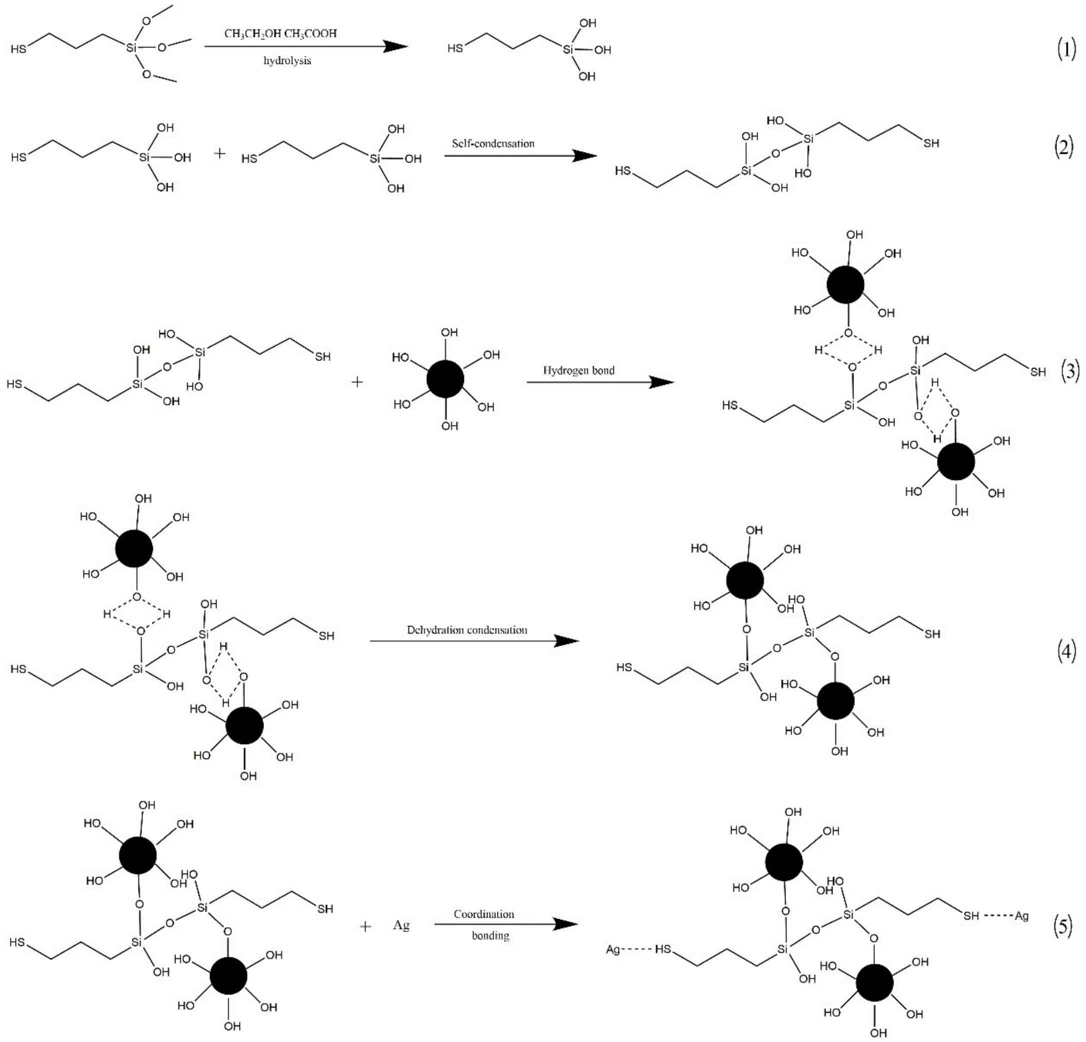


Fig. S2 Schematic of the reaction between each component.

Table S1 Summary of the performance of stretchable conductive composites.

| Filler | Matrix | Conductivity (S/m) | Electrical properties | Application | Ref. |
|---|---|---|---|---|---|
| MWCNT | SR | ~1 | - | - | 2 |
| MWCNT forest | PU | 50-100 | Almost constant for strain up to 40% | - | 40 |
| MWCNT | PDMS | $10^{-3}$ | Decrease with the strain | - | 9 |
| CuNW-PVA | PDMS | 83 | Almost stable within 60% strain | - | 42 |
| Carbon fiber | PDMS | 20-40 | Almost stable within 80% train | Stretchable interconnects | 41 |
| MWCNT-PDA-Ag | SR foam | 5 | - | Electromagnetic shielding | 48 |
| SMCB@Ag | SR | 10 | Stable within 100% strain | Stretchable conductor | This work |
